# Delineation of Early and Later Adult Onset Depression by Diffusion Tensor Imaging

**DOI:** 10.1371/journal.pone.0112307

**Published:** 2014-11-13

**Authors:** Yuqi Cheng, Jian Xu, Hongjun Yu, Binbin Nie, Na Li, Chunrong Luo, Haijun Li, Fang Liu, Yan Bai, Baoci Shan, Lin Xu, Xiufeng Xu

**Affiliations:** 1 Department of Psychiatry, First Affiliated Hospital of Kunming Medical University, Kunming, PR China; 2 Department of Internal Medicine, First Affiliated Hospital of Kunming Medical University, Kunming, PR China; 3 Magnetic Resonance Imaging Center, the First Hospital of Kunming City, Kunming, PR China; 4 Key Laboratory of Nuclear Analysis Techniques, Institute of High Energy Physics, Chinese Academy of Sciences, Beijing, PR China; 5 Key Laboratory of Animal Models and Human Disease Mechanisms, Chinese Academy of Sciences & Yunnan Province, Kunming Institute of Zoology, Kunming, PR China; Shanghai Mental Health Center, Shanghai Jiao Tong University School of Medicine, China

## Abstract

**Background:**

Due to a lack of evidence, there is no consistent age of onset to define early onset (EO) versus later onset (LO) major depressive disorder (MDD). Fractional anisotropy (FA), derived from diffusion tensor imaging (DTI), has been widely used to study neuropsychiatric disorders by providing information about the brain circuitry, abnormalities of which might facilitate the delineation of EO versus LO MDD.

**Method:**

In this study, 61 pairs of untreated, non-elderly, first-episode MDD patients and healthy controls (HCs) aged 18–45 years old received DTI scans. The voxel-based analysis method (VBM), classification analysis, using the Statistical Package for the Social Sciences (SPSS), and regression analyses were used to determine abnormal FA clusters and their correlations with age of onset and clinical symptoms.

**Results:**

Classification analysis suggested in the best model that there were two subgroups of MDD patients, delineated by an age of onset of 30 years old, by which MDD patients could be divided into EO (18–29 years old) and LO (30–45 years old) groups. LO MDD was characterized by decreased FA, especially in the white matter (WM) of the fronto-occipital fasciculus and posterior limb of internal capsule, with a negative correlation with the severity of depressive symptoms; in marked contrast, EO MDD showed increased FA, especially in the WM of the corpus callosum, corticospinal midbrain and inferior fronto-occipital fasciculus, while FA of the WM near the midbrain had a positive correlation with the severity of depressive symptoms.

**Conclusion:**

Specific abnormalities of the brain circuitry in EO vs. LO MDD were delineated by an age of onset of 30 years old, as demonstrated by distinct abnormal FA clusters with opposite correlations with clinical symptoms. This DTI study supported the evidence of an exact age for the delineation of MDD, which could have broad multidisciplinary importance.

**Trial Registration:**

ClinicalTrials.gov NCT00703742

## Introduction

Major depressive disorder (MDD) is well known for its clinical heterogeneity, including variability in the age of onset, constellations of depressive symptoms, and disease severity and course [1]. This heterogeneity has been believed to be one of the greatest barriers to successful treatment of MDD because it has been assumed to be attributable to different etiologies and pathophysiologies [2], for which MDD patients would require different treatments, and based on which patients would have different responses to the same treatment. Unfortunately, evidence is still lacking for defining the heterogeneity of MDD.

Genetic studies might have provided molecular evidence for the heterogeneity of MDD. Twin studies have estimated the average heritability of MDD at approximately 40% [3], but it this rate increased to 47% in early onset (EO, <30 years old), while it decreased to only 10% in later onset (LO, >30 years old) [4]. Furthermore, the heritability of MDD was found to be significantly lower as a function of the age of onset, at 50% in first-degree relatives when the proband's age of onset was younger than 20 years old but 36% when probands were in their 20 s, 25% when they were in their 30 s and 16% when they were older than 40 years old [5]. These genetic studies suggested that EO (<30 years old) and LO (>30 years old) MDD possibly have different etiologies. Moreover, a large-scale genetic association study recruited MDD patients with age of onset younger than 30 years old as EO patients vs. LO, and EO was found to be associated with disease severity and the chronicity of depressive symptoms [6]. Nevertheless, the delineative age of onset for EO vs. LO has been highly inconsistently reported in different studies, in which an ages of onset younger than18 [7], 25 [8], 30 [9] or 45 [10] years old have all been used to define EO vs. LO MDD. Thus far, due to a lack of evidence, there is no generally agreed upon age of onset to define EO vs. LO MDD.

An increasing number of studies have supported the theory of dysfunction of the whole brain network in MDD. The brain circuitry might be a good candidate for delineating EO from LO MDD because it is believed to be an intermediate step between molecular and behavioral levels. The white matter (WM) contains neuronal fibers that connect neurons in the network of the brain. MDD patients showed reduced density and number of glial cells in the anterior cingulate cortex [11]. Altered density and ultrastructure of oligodendrocytes were also detected in the prefrontal cortex and amygdala of MDD patients [12]. Nonintrusive brain imaging techniques have been widely used to study neuropsychiatric disorders clinically [13]. In particular, fractional anisotropy (FA), derived from diffusion tensor imaging (DTI), has been widely used to quantify neural tracts [14], abnormalities of which could cause EO or LO MDD, based on specific information about the brain circuitry [15], which could connect evidence on the molecular and behavioral levels.

Imaging studies have revealed global or regional WM abnormalities in depression, but significant differences between EO and LO depression suggest different etiological mechanisms [16]. Widespread FA abnormalities, especially in the frontal and temporal lobes, have been found to be associated with late-life MDD [17]–[19] and with disease outcomes [20]. These abnormalities might also present in non-elderly patients or in the early course of MDD [21], and they have been associated with special executive dysfunction [22] and poor response to antidepressant treatment [23]. However, highly variable location of clusters with FA abnormalities in MDD patients has been reported, and both increased and decreased FA have been reported following treatment with antidepressants [23], [24]. Perhaps these inconsistencies could be at least partially attributed to the heterogeneity of MDD. Recently, a DTI study was conducted to differentiate bipolar depression from unipolar depression [25], raising the possibility of classifying MDD via a DTI technique. Because the sensitivity of dendrites to stress [26] and age-dependent FA changes were discovered [27], FA value could be developed as good indicators, reflecting the possible differences between MDD patients with different ages of onset. Nevertheless, as variable constellations of depressive symptoms, differences in responses to antidepressants, in age of onset and in severity of course could compromise the study of and individual medication treatment for MDD, so the classification of MDD, based on both clinical data and biomarkers, such as regional FA values, should be a useful method for these purposes. To examine this possibility, we recruited sixty-one pairs of untreated non-elderly, first-episode MDD patients and healthy controls (HCs) aged 18–45 years old to exclude possible interferences, such as drug treatment, recurrent episodes and vascular problems in older age (>45 years old).

## Materials and Methods

### Subjects

This research was approved by the Institutional Review Board of Kunming Medical University, Yunnan Province, P. R. China (ClinicalTrials.gov: NCT00703742). The protocol for this trial and supporting checklist are available as supporting information; see Checklist S1 and Protocol S1. Each participant was required to sign a written informed consent form after receiving a complete description of the study. All of the participants were recruited from June 2008 to July 2011.

The diagnosis of MDD was independently made by two experienced psychiatrists in accordance with the Diagnostic and Statistical Manual of Mental Disorders, Fourth Edition (DSM-IV, American Psychiatric Association, 1994). A total of 77 right-handed MDD patients were recruited from among the out/inpatients of the Department of Psychiatry, the First Affiliated Hospital of Kunming Medical University. Two MDD patients refused to sign written informed consent forms, and another two MDD patients signed written informed consent form but did not participate in the study. Thus, 73 MDD patients underwent clinical screening (Figure S1 in File S1). These MDD patients were Han Chinese people between 18 and 45 years old, and had scores of 17 or greater on the 17-item Hamilton Depression Rating Scale (HDRS), and were untreated and in their first episodes. Data on age, age of onset, duration of disease, sex and years of education were collected. The duration of disease was less than 2 years, defined as the initial manifestation clearly attributable to MDD until the day of acquisition of magnetic resonance imaging (MRI).

The exclusion criteria included the following items: (1) another diagnosed axis I psychiatric disorder; (2) organic brain disorders or neurological disorders; (3) obvious psychiatric symptoms, such as delusions or hallucinations; (4) any physical illness as assessed by personal history; (5) clinical conditions that could cause cerebral atrophy (such as a history of arterial hypertension, diabetes mellitus, stroke); and (6) inability to undergo an MRI scan, including subjects with metal implants. Of these MDD patients who participated in clinical screening, only 68 MDD patients met the exclusion criteria and thus underwent MRI scans.

Matched by sex, age and years of education with the MDD patients on one-to-one basis, 65 right-handed Han Chinese healthy controls (HCs) were recruited by an advertisement in the local community. All of the HCs were free of histories of any psychiatric, neurological or organic diseases according to the exclusion criteria and were without known histories of any psychiatric illnesses in first-degree relatives.

The depressive symptoms and anxiety of all of the participants were assessed by an experienced psychiatrist using the 17-item Hamilton Depression Rating Scale (HDRS, Hamilton 1960) and the 14-item Hamilton Anxiety Scale (HAMA).

### Image acquisition

MRI sequences were performed by a skilled radiological technician on a 1.5-T clinical GE MRI scanner (Twinspeed, Milwaukee, WI, USA) equipped with a birdcage head coil. Restraining foam pads were used to minimize head motion. All of the participants underwent a normal T1 scan to detect obvious structural abnormalities, and the parameters were as follows: repetition time (TR)/echo time (TE)  = 1800/8.9 ms; matrix size  = 256×256; slice thickness  = 5 mm; gap  = 1 mm; and field of view  = 240 mm.

Diffusion tensor imaging (DTI) was acquired using a diffusion tensor echo planar pulse sequence, and the parameters were as follows: TR/TE  = 12,000/89 ms; matrix size  = 128×128; slice thickness  = 6 mm with no interslice gap; field of view  = 240 mm; and number of excitations  = 5. The diffusion sensitizing gradients were applied in 13 non-collinear directions (b = 1000 s/mm^2^), together with acquisition without diffusion weighting (b = 0). Twenty-four contiguous slices were acquired to cover the entire brain. All of the sections were acquired parallel to the anterior-posterior commissure line.

### Data preprocessing

All of the images were transferred to an IBM workstation in DICOM format. For each participant, all of their DTI images with b0 images were analyzed using DTI-Studio software (https://www.dtistudio.org/, version 2.3; H. Jiang, S. Mori; the Johns Hopkins University, Baltimore, MD, USA), and parametric images with FA were generated. The FA images and b0 images were saved in ANALYZE format. Statistical Parametric Mapping software (SPM5, Wellcome Department of Cognitive Neurology, London, UK) was used to normalize the b0 images to the standard Montreal Neurological Institute (MNI) space and to resample them at a final voxel size of 1×1×1 mm^3^. The transformation matrix was applied to the FA map to normalize the FA map to the standard MNI space. Before statistical analysis, the normalized FA maps were smoothed by using an 8-mm full-width half-maximum Gaussian kernel.

### Identification of differences between MDD patients and HC

The two-sample t test was performed in a voxel-by-voxel manner using SPM5, to detect abnormal FA clusters from the whole brain between the MDD patients and HCs. A threshold of p<0.05 after small volume correction was considered to be statistically significant.

### FA value retrieval and classification analysis

The abnormal FA clusters, identified using SPM5, were set as the regions of interest (ROIs), and the mean FA value of each ROI was calculated for each individual. Using classification analysis in the Statistical Package for the Social Sciences 17.0 (SPSS, Inc., Chicago, IL, USA), the mean FA value of each ROI and the age of onset were analyzed as follows: (1) two-step cluster analysis identified the best numbers of category of subgroups using the Schwarz Bayesian Criterion (BIC); and (2) K-means cluster analysis identified each individual in the subgroups. All the FA data of these ROIs were standardized before clustering.

### Characterization of different subgroups

According to the results revealed by classification analysis in SPSS, the MDD patients and their paired HCs were divided into 2 subgroups. Then, the two-sample t test (SPM5) was used to determine abnormal FA clusters from the whole brain between the MDD patients and HCs in each subgroup, and multiple regression analysis (SPM5) was used to detect the associations between FA map and HDRS scores. All of the results were considered to be statistically significant at a level of p<0.05 after the corrections described above.

## Results

### Clinical data

Sixty-eight MDD patients who met the inclusion and exclusion criteria received DTI scans. Seven cases were discarded because of brain structural abnormalities detected by T1 weighted MRI (4 cases with focal ischemia in the frontal lobe or basal ganglia, 2 cases with focal infarctions in the left parietal lobe and 1 case with a brain cyst in the right occipital lobe). Sixty-five healthy controls (HCs) also received DTI scans. Four cases were discarded for similar reasons (3 cases with focal ischemia in the frontal lobe and 1 case with a brain cyst in the basal ganglia). In total, sixty-one pairs of MDD patients and HCs were used for further analyses (Figure S1 in File S1).

There were no group differences in age, years of education and sex ratio (17 men and 44 women) between the sixty-one pairs of MDD patients and the HCs (Table S1 in File S1). The sixty-one MDD patients showed a mean duration of disease of 12.34±8.40 months, and the total scores on the HDRS and HAMA were 22.43±3.86 and 17.03±6.36, respectively (Table 1).

**Table 1:** Demographic data and FA value of three clusters with difference between MDD and HCs.

*Variable*	*HCs (n = 61)*	*MDD (n = 61)*		*t/χ^2^*	*P value*
	*Mean (SD)*	*Mean (SD)*			
**Sex (male/female)**	**17/44**	**17/44**		**–**	**–**
**Age (year)**	**29.56 (7.06)**	**29.02 (7.97)**		**0.397**	**0.692**
**Education (year)**	**13.16 (2.79)**	**12.93 (3.00)**		**0.437**	**0.663**
**HDRS score**	**2.38 (2.18)**	**22.70 (3.80)**		**−36.249**	**0.000**
**HAMA score**	**2.37 (2.27)**	**17.00 (6.39)**		**−16.832**	**0.000**
**FA value of RTEMP**	**0.3196 (0.0251)**	**0.3032 (0.0288)**		**3.344**	**0.001**
**FA value of RMFG**	**0.2212 (0.0287)**	**0.2074 (0.0219)**		**2.997**	**0.003**
**FA value of LOCG**	**0.2042 (0.0236)**	**0.2173 (0.0232)**		**−3.087**	**0.003**
		***EO (n = 32)***	***LO (n = 29)***		
**Sex (male/female)**	**–**	**10/22**	**7/22**	**0.383**	**0.536**
**Age (year)**	**–**	**22.38 (3.60)**	**36.35 (3.96)**	**−14.457**	**0.000**
**Age of onset (year)**	**–**	**21.22 (3.61)**	**35.14 (3.87)**	**−14.537**	**0.000**
**Education (year)**	**–**	**12.78 (2.85)**	**13.10 (3.20)**	**−0.416**	**0.679**
**HDRS score**	**–**	**22.97 (4.21)**	**22.41 (3.34)**	**0.577**	**0.573**
**HAMA score**	**–**	**17.38 (7.23)**	**16.59 (5.42)**	**0.478**	**0.634**
**Duration (month)**	**–**	**12.35 (9.56)**	**11.62 (6.95)**	**0.338**	**0.736**
**FA value of RTEMP**		**0.3037 (0.0299)**	**0.3026 (0.0281)**	**0.140**	**0.889**
**FA value of RMFG**		**0.2043 (0.0195)**	**0.2109 (0.0241)**	**−0.281**	**0.246**
**FA value of LOCG**		**0.2157 (0.0229)**	**0.2190 (0.0238)**	**−0.861**	**0.586**

RTEMP, RMFG and LOCG represent significant clusters in MDD differed from HCs (showed in Figure S2 in File S1). Abbreviations: MDD, major depressive disorder; HCs, healthy control; HDRS, Hamilton depression rating scale; HAMA, Hamilton anxiety scale; EO, early onset depression; LO, later onset depression. RTEMP, right temporal lobe; RMFG, right middle frontal lobe; LOCG, left occipital lobe.

### MDD subgroups

**Figure 1:**
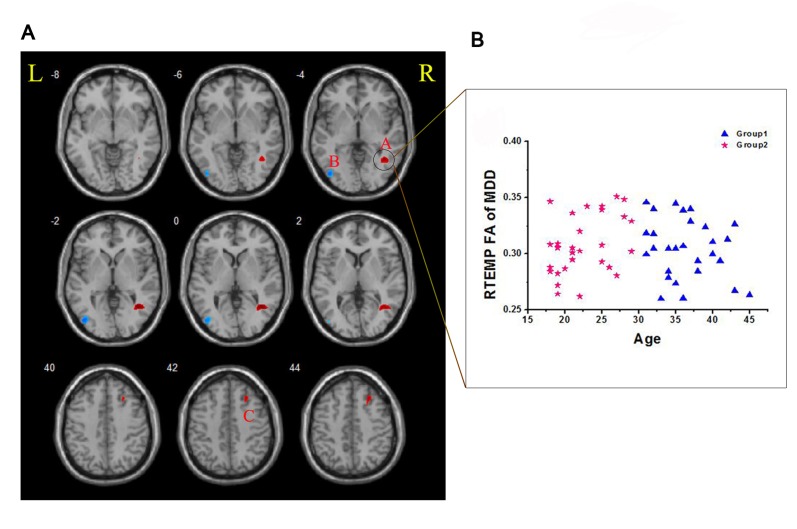
Classification analysis based on the differences in FA between MDD patients and HCs. A. Clusters of red color indicate significant increases in FA values, while those of blue color show decreases in FA values in MDD patients, compared with HCs. B. FA values of the cluster in each individual were classified into two groups, indicated by red (EO) and blue (LO) points plotted against age. L, left side; R, right side; a, cluster near right temporal lobe; b, cluster near left occipital lobe; c, cluster near right middle frontal lobe; MDD, major depressive disorder; HCs, healthy controls; RTEMP, right temporal lobe.

A voxel-based analysis method (VBM) for diffusion tensor imaging (DTI) was used to identify abnormal FA clusters in MDD patients vs. HCs. When sixty-one pairs of participants between 18 and 45 years old were collected, we found a pattern of two clusters with decreased FA located in the right temporal lobe (RTEMP) and right middle frontal lobe (RMFG), while one cluster with increased FA was in the left occipital lobe (LOCG) (Figure 1A). We suspected that this result might have been only the average effect of MDD without considering different ages of onset in the effects on different patterns of significant FA clusters.

To address this possibility, the clusters RTEMP, RMFG and LOCG were set as the regions of interest (ROIs), and their FA values were retrieved from each individual. Using the FA value of these three ROIs, two-step classification analysis in SPSS17.0 software revealed that the best number of categories (subgroup) for MDD patients was two (Table S1 in File S1), and K-mean cluster analysis categorized each individual into one of the two subgroups. We found that the age ranges of the onset for the two subgroups were 18–29 and 30–45 years old (Table S2 in File S1), which was surprisingly consistent with the definition of EO vs. LO in certain genetic association studies. Due to the relatively short durations of depression in the present samples, the age (actual age when patients received the MRI scans) ranges for the 2 subgroups were 18–29 and 30–45 years old also (Table S2 in File S1). To understand this best model further, we created a scatter plot of FA values for RTEMP against age (Figure 1B), in which a clear borderline at 30 years old for these two groups was found.

### Abnormal FA clusters

Accordingly, the sixty-one pairs of participants were divided into two subgroups, of which the EO group consisted of thirty-two pairs aged 18–29 years old, and the LO group had twenty-nine pairs aged 30–45 years old. The EO and LO MDD patients had no differences in mean score on the HDRS (t = 0.042, p = 0.967) or mean duration of disease (t = 0.090, p = 0.928). However, we found that EO and LO MDD patients indeed were the major contributors of abnormal clusters with increased and decreased FA, respectively.

**Figure 2:**
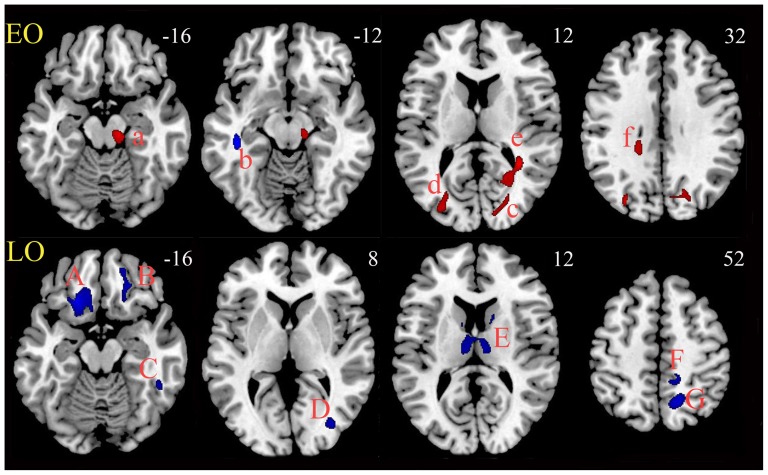
FA clusters for EO vs. LO MDD. Upper panel (EO): Five clusters (a, c–f) with increased FA (red color) but one cluster (b) with decreased FA (blue color) was found in EO patients, compared with the paired HCs. Lower panel (LO): seven clusters (A–G), all with decreased FA (blue color), were found in LO patients compared with the paired HCs. EO, early onset; LO, later onset; MDD, major depressive disorder; HCs, healthy controls.

In EO MDD patients, compared with their paired HCs, decreased FA values were found only in the left inferior longitudinal fasciculus (near the parahippocampal gyrus and superior temporal gyrus), wherever increased FA was identified in vast areas, including the bilateral corpus callosum (near the posterior cingulate gyrus), left inferior fronto-occipital fasciculus (superior occipital gyrus), right forceps major and optical radiation (cuneus, middle occipital gyrus) and right corticospinal midbrain (near the substantia nigra) (Figure 2; Table S3 in File S1).

In marked contrast, in LO MDD patients, compared with their paired HCs, all seven of the clusters showed decreased FA, and their locations were much different from those of the clusters in the EO MDD patients. The seven clusters were in the white matter in the bilateral inferior fronto-occipital fasciculus (near the bilateral orbitales gyrus), left posterior limb of the internal capsule (near the caudate), right posterior corona radiate (near the precunus), right inferior longitudinal fasciculus (inferior temporal gyrus), right superior thalamic radiation (near the cingulated gyrus) and inferior fronto-occipital fasciculus (near the middle occipital lobe) (Figure 2; Table S3 in File S1).

To confirm further whether EO and LO MDD were the major contributors to increased and decreased FA clusters, respectively, we hypothesized that there was a transient period, such as 26–29 years old, between EO and LO, and if the samples within this period were removed, the increased and decreased FA clusters for EO and LO MDD, respectively, would become clear-cut. Remarkably, after removal of MDD patients with ages of onset 26–29 years old (7 subjects), as well as the paired HCs, we found that EO MDD patients (18–25 years old, 25 subjects) now had abnormal clusters all with increased FA only (Table S4 and Figure S2 in File S1), which was in marked contrast to the LO MDD patients (30–45 years old), who showed abnormal clusters, all with decreased FA only. Thus, the delineation of EO and LO MDD at the age of onset of 30 years old, with 26–29 years old as a transient period, revealed distinct characteristics of the brain circuitry consistent with the assumption that EO and LO MD are attributable to different etiologies and pathophysiologies.

### Correlation with clinical symptoms

It is believed that the brain circuitry, as an intermediate step, can be used as a substitute for both molecular and clinical meaningful observations. Because EO and LO MDD < delineated by the age of onset on 30 years old, have different heritability, we expected that this delineation of EO and LO MDD would also have distinct correlations with clinical symptoms, such as HDRS scores. Using multiple regression analyses of SPM5, we found that EO and LO MDD patients indeed showed distinct correlations with HDRS scores.

**Figure 3:**
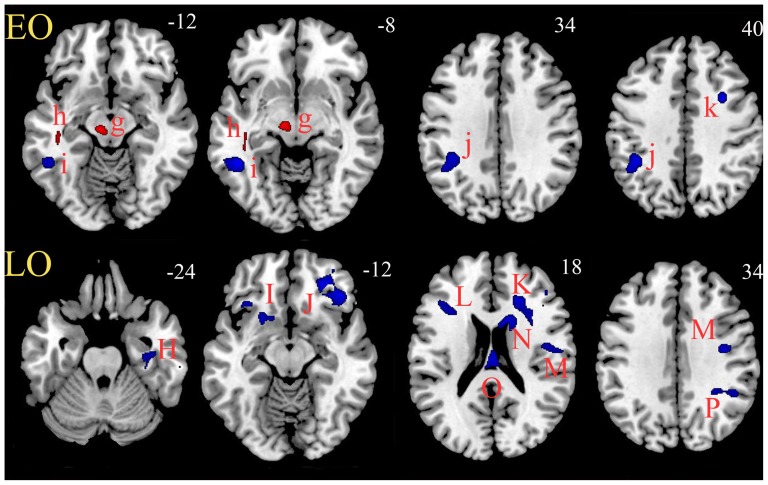
FA clusters with correlations with HDRS scores for EO vs. LO MDD. Upper panel (EO): two FA clusters (g, h) with positive correlations (red color) and three FA clusters (i, j, k) with negative correlations (blue color) with HDRS scores. Lower panel (LO): nine FA clusters (H-P), all with negative correlations (blue) with HDRS scores. EO, early onset; LO, later onset; MDD, major depressive disorder; HDRS, Hamilton Depression Rating Scale.

In EO MDD patients, both positive and negative correlations were identified between the total HDRS score and the FA values of some regions. Positive correlations were found in the left corticospinal/corticopontine tract in midbrain (near the substantia nigra) and inferior longitudinal fasciculus (near the fusiform gyrus and parahippocampal gyrus) (red color in Figure 3; see also Table S5 in File S1). Negative correlations were found in the left inferior longitudinal fasciculus (near the middle temporal gyrus), superior longitudinal fasciculus (near the inferior parietal lobule) and right superior longitudinal fasciculus (near the middle frontal gyrus) (Figure 3; Table S5 in File S1).

In marked contrast, in the LO MDD patients, only negative correlations were found between all nine clusters and HDRS scores. These regions were the left inferior fronto-occipital fasciculus (near the orbitales gyrus), right uncinate fasciculus/inferior fronto-occipital fasciculus (near the orbitales gyrus), right anterior corona radiata/superior fronto-occipital fasciculus (near the inferior frontal gyrus), left eternal capsule/inferior fronto-occipital fasciculus (near the insula), right superior longitudinal fasciculus (near the precentral gyrus), left fornix (near the thalamus), right anterior limb of internal capsule (near the caudate), right cingulum (near the parahippocampal gyrus) and right posterior corona radiata/superior longitudinal fasciculus (near the precuneus) (Figure 3; Table S5 in File S1).

Finally, when we considered the clusters with both abnormal FA and correlations with clinical symptoms, the characteristics of the brain circuitry for EO and LO MDD patients became more clear. In LO MDD, some regions with decreased FA values overlapped with regions correlated with the depressive severity. FA values in the cluster in the bilateral uncinate fasciculus/inferior fronto-occipital fasciculus near the orbital frontal lobe decreased obviously wherever they were negatively correlated with HDRS score. For the fornix/posterior limb of the internal capsule near the posterior cingulate gyrus, FA values also decreased obviously in MDD patients and were negatively correlated with HDRS scores. In EO MDD, the FA values of the cluster in the left inferior longitudinal fasciculus near the parahippocampal gyrus decreased obviously wherever they were positively correlated with HDRS scores (Figure S3 in File S1).

## Discussion

In the present study, we demonstrated the novel finding that specific DTI evidence about information regarding the brain circuitry, with distinct correlations with clinical symptoms, supported the delineation of EO versus LO MDD in non-elderly patients, based on an age of onset of 30 years old. This finding was consistent with the distinct heritability of EO (<30 years old) vs. LO MDD (>30 years old) reported in certain genetic association studies. Thus, there might be at least two different groups in non-elderly MDD: one with a younger age of onset and characterized by stronger WM connectivity; and another with an older age of onset but characterized by WM microstructural deficits. Even with similar clinical manifestations, these two groups might have different neurological pathologies.

The regions with decreased FA for LO were mainly located in the WM near the orbital frontal cortex (OFC) and thalamus. The FA values of these regions were also negatively correlated with severity of depression, suggesting that the WM deficits of these areas constituted the main pathophysiology of LO MDD. Alterations in the functional balance between the OFC and the circuits it forms with related areas of the temporal lobe, striatum, thalamus, and brain stem might underlie the pathophysiology of MDD [28]. As a key structure inducing the stress response in the body, the thalamus has been believed to underlie emotional distress in humans [29] and was found to be related to depression [30]. A study in animals also confirmed that older adults responded to stress with greater increases in plasma levels of adrenocorticotropic hormone and modest reductions in glucocorticoid feedback sensitivity, compared to young adults [31]. Thus, these results might reflect the possibility of greater stress effects in the LO MDD group than in the EO MDD group.

Unlike in LO MDD, the FA values increased in brain regions near the frontal, parietal and cingulate areas in the EO group. Although most articles have reported decreased FA in MDD patients, contrary results were also reported recently. MDD patients who did not remit after sertraline treatment exhibit higher FA values in the superior frontal gyri and anterior cingulate cortices [24]. Wildly increased FA has also been reported in obsessive compulsive disorder patients, including in the regions near the subinsular cortex [32], the cingulum and the anterior limb of the internal capsule [33], and the corpus callosum [34]. These increased regional FA values could be restored by antidepressants [34]. Until now, the essential association of FA with mental disorders has not been fully understood yet. However, it has been suggested that the higher FA might be related to an increase in the connectivity of WM bundles [35]. In schizophrenia, higher FA in the anterior corpus callosum was found in individuals with symptoms of hallucinations [36]. These results suggested that higher FA in some regions might have reflected strengthened connectivity and might have been related to special characteristics, such as recurrence, in the EO group [37]. However, for the LO group, the main pathology might be deficits in WM microstructural integrity.

The present finding that specific DTI abnormalities with distinct correlations with clinical symptoms supported the delineation of EO and LO MDD based on the age of onset of 30 years old could have significant impact on multidisciplinary treatment for MDD. First, due to a lack of evidence, there is no generally agreed upon age of onset to define EO and LO MDD. Second, a recent report suggested that DTI abnormalities could reflect genetic effects because the S-allele carriers of serotonin transporter polymorphisms had lower FA in the frontolimbic areas than L-allele homozygotes [38]. Thus, the present finding of a delineation of EO and LO MD based on the age of onset of 30 years old might also reflect genetic effects. Third, because the heritability of MDD is different for EO and LO patients [4], it is possible that DTI abnormalities for EO and LO of MDD might be attributable to differences in genetic effects and environmental factors [39], [40]. Fourth, a large-scale study of DSM-IV disorders found that the lifetime prevalence of MDD was variable with different ages of onset, e.g., 15.4% for EO (18–29 years old) and 19.8% for LO (30–44 years old) [41]. Thus, the delineation of EO and LO MDD by an age of onset of 30 years old was supported by the present DTI evidence and also the evidence from previous molecular and clinical studies.

Because MDD is highly heterogeneous, it is difficult to find “common” clusters with abnormal FA in all individuals, particularly when the sample size is large and the range of ages or ages of onset is broad. As variable constellations of depressive symptoms, differences in responses to antidepressants, age of onset and severity of course could compromise the study of and individual medication treatment for MDD, so the classification of subtypes, based on both clinical data and biomarkers, such as regional FA values, could constitute a useful method for these purposes.

## Supporting Information

Checklist S1 **TREND checklist.**
(PDF)

File S1 **Supporting files.** **Figure S1**, Procedure of recruiting and data collection. **Figure S2**, Only increased FA was found in MDD patients at the age of 18–25 years old compared with paired HCs. **Figure S3**, Clusters correlated to HDRS (Hamilton depression rating scale). **Table S1**, Results of Two-step classification using the FA value of three ROIs of RTEMP, RMFG and LOCG. **Table S2**, Result of K-mean cluster classify analysis in MDD. **Table S3**, Abnormal FA clusters in EO and LO subgroups of MDD. **Table S4**, Abnormal FA clusters in MDD patients at the age of 18–25 years old compared with paired HCs. **Table S5**, FA Clusters correlated with HDRS scores in EO and LO subgroups of MDD.
(DOC)

Protocol S1 **Trial protocol.**
(DOCX)
