# Supplementary material for: Delineation of Early and Later Adult Onset Depression by Diffusion Tensor Imaging
Source: PLoS One. 2014 Nov 13;9(11):e112307. doi: 10.1371/journal.pone.0112307 (PMC4231105; doi:10.1371/journal.pone.0112307)
Supplement: File S1 — Supporting files. Figure S1, Procedure of recruiting and data collection. Figure S2, Only increased FA was found in MDD patients at the age of 18–25 years old compared with paired HCs. Figure S3, Clusters correlated to HDRS (Hamilton depression rating scale). Table S1, Results of Two-step classification using the FA value of three ROIs of RTEMP, RMFG and LOCG. Table S2, Result of K-mean cluster classify analysis in MDD. Table S3, Abnormal FA clusters in EO and LO subgroups of MDD. Table S4, Abnormal FA clusters in MDD patients at the age of 18–25 years old compared with paired HCs. Table S5, FA Clusters correlated with HDRS scores in EO and LO subgroups of MDD. (DOC) [file pone.0112307.s002.doc]

**Supplementary materials**

**Figure S1 Procedure of recruiting and data collection.**

Recruiting,

MDD (n = 77), HCs (n = 65)

T1, T2 structural MRI scaning,

MDD ( n = 68), HCs (n = 65)

DTI scan,

MDD (n = 63), HCs (n = 61)

Screening,

MDD (n = 73), HCs (n = 65)

We excluded 5 cases in MDD group throughout personal history and physical examination (2 for hypertension, 2 for history of head trauma, 1 for history of thyroid disease).

We excluded 5 cases in MDD group for obvious structural abnormality (2 for focal ischemia, 2 for focal infarction, 1 for cyst). Four cases in HCs group were excluded for focal ischemia and cyst.

Two patients refused to sign the informal consent.

Explaining study and informal consent,

MDD (n = 75), HCs (n = 65)

Two patients did not come.

Analyses ,

MDD (n = 61), HCs (n = 61)

Two patients were excluded after the follow-up investigation one year after. one patient for mania symptom and one for psychiatric symptom.

**Figure S2**


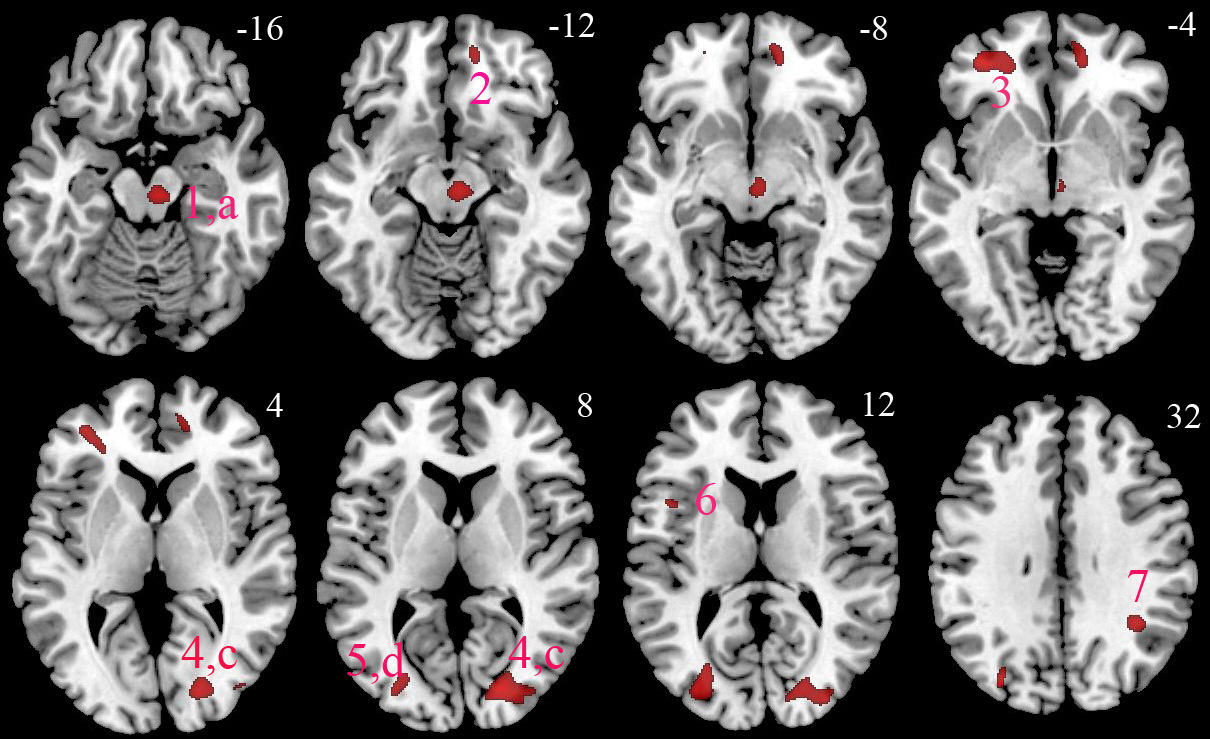


**Only increased FA was found in MDD patients at the age of 18-25 years old compared with paired HCs.** Younger patients (18-25 years old) showed abnormal clusters with increased FA only, while EO subgroup (18-29 years old) showed abnormal clusters with either increased or decreased FA. In marked contrast, LO subgroup (30-45 years old) exhibited abnormal clusters with decreased FA only. Number 1 to 7: clusters listed in supplementary table 3; Small letters a, c, d: clusters showed in EO panel of figure 2 and listed in supplementary table 2.

**Figure S3**


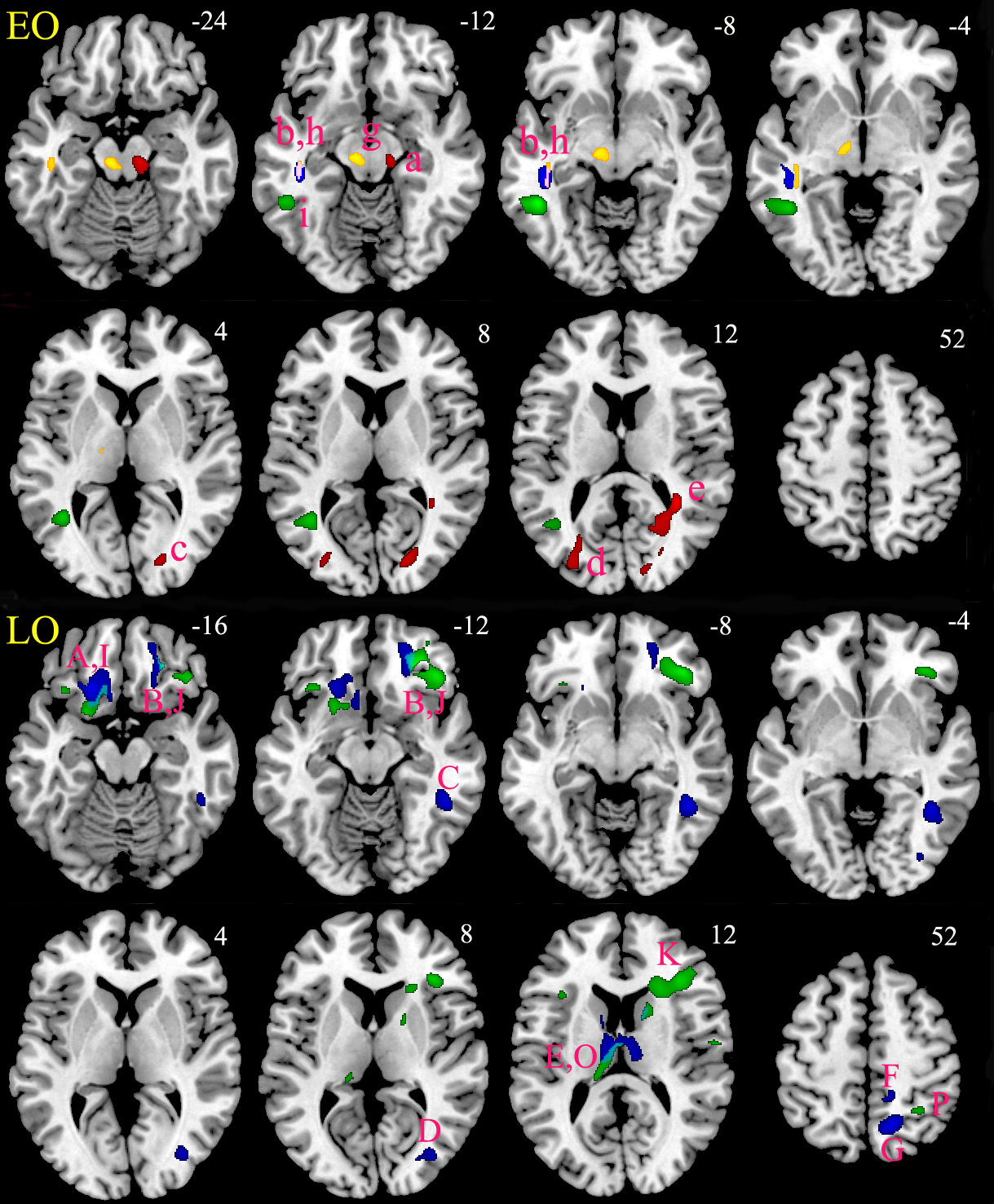


**Clusters correlated to HDRS (Hamilton depression rating scale).** Panel EO. Cluster a indicate decrease of FA value in EO subgroup compared with paired HCs and cluster h indicate FA value with positive correlation to HDRS. Clusters b and h were overlapped in location, suggesting that this abnormality may serve as a DTI marker linking clinical symptom of EO subgroup. Panel LO. Clusters A, B and E indicate decrease of FA value in LO subgroup compared with paired HCs, and the clusters I, J and M indicate FA value with negative correlation to HDRS. Clusters A and I, B and J, and E and O were overlapped in location, respectively. These results suggest that these abnormalities may serve as DTI markers linking clinical symptom of LO subgroup. Red color, increased FA in MDD; blue color, decreased FA in MDD; yellow color, positive correlation of FA value to HDRS; green color, negative correlation of FA value to HDRS.

**Table S1 Results of Two-step classification using the FA value of three ROIs of RTEMP, RMFG and LOCG.**

| **Clusters** | **Schwarz's Bayesian Criterion (BIC)** | **BIC Change** | **Ratio of BIC Changes** | **Ratio of Distance Measures** |
| --- | --- | --- | --- | --- |
| **1** | **150.005** |  |  |  |
| **2** | **145.080** | **-4.925** | **1.000** | **1.477** |
| **3** | **149.717** | **4.638** | **-.942** | **1.707** |
| **4** | **162.652** | **12.935** | **-2.626** | **1.646** |
| **5** | **180.191** | **17.539** | **-3.561** | **1.097** |
| **6** | **198.358** | **18.168** | **-3.689** | **1.064** |
| **7** | **216.914** | **18.556** | **-3.767** | **1.445** |
| **8** | **237.351** | **20.437** | **-4.149** | **1.166** |
| **9** | **258.389** | **21.038** | **-4.271** | **1.091** |
| **10** | **279.728** | **21.339** | **-4.333** | **1.257** |
| **11** | **301.748** | **22.020** | **-4.471** | **1.123** |
| **12** | **324.058** | **22.310** | **-4.530** | **1.017** |
| **13** | **346.408** | **22.350** | **-4.538** | **1.022** |
| **14** | **368.808** | **22.400** | **-4.548** | **1.015** |
| **15** | **391.241** | **22.433** | **-4.555** | **1.069** |

**Table S2 Result of K-mean cluster classify analysis in MDD**

| **Case** | **Code** | **Age** | **Age of onset** | **FA value of**  **RTEMP** | **FA value of**  **RMFG** | **FA value of**  **LOCG** | **Classify result** | **Distance from the center** |
| --- | --- | --- | --- | --- | --- | --- | --- | --- |
| **1** | **D001** | **35** | **33** | **0.2931** | **0.1811** | **0.2177** | **1** | **1.346** |
| **2** | **D002** | **25** | **24** | **0.2791** | **0.1905** | **0.1986** | **2** | **2.719** |
| **3** | **D005** | **34** | **33** | **0.3180** | **0.2566** | **0.2306** | **1** | **2.345** |
| **4** | **D006** | **32** | **31** | **0.2843** | **0.2353** | **0.2050** | **1** | **4.345** |
| **5** | **D007** | **38** | **38** | **0.3290** | **0.1697** | **0.2037** | **1** | **1.656** |
| **6** | **D008** | **37** | **35** | **0.3390** | **0.2315** | **0.2441** | **1** | **0.657** |
| **7** | **D009** | **36** | **35** | **0.2825** | **0.1886** | **0.2247** | **1** | **0.348** |
| **8** | **D011** | **19** | **19** | **0.3394** | **0.2055** | **0.1515** | **2** | **3.281** |
| **9** | **D013** | **25** | **23** | **0.3266** | **0.1935** | **0.2229** | **2** | **2.720** |
| **10** | **D019** | **43** | **41** | **0.2620** | **0.2122** | **0.1768** | **1** | **6.655** |
| **11** | **D023** | **22** | **22** | **0.3047** | **0.2366** | **0.2286** | **2** | **0.287** |
| **12** | **D028** | **35** | **35** | **0.3467** | **0.2177** | **0.2270** | **1** | **1.345** |
| **13** | **D029** | **18** | **17** | **0.3026** | **0.2072** | **0.2342** | **2** | **5.281** |
| **14** | **D031** | **22** | **21** | **0.2643** | **0.1991** | **0.1898** | **2** | **0.282** |
| **15** | **D034** | **19** | **19** | **0.3334** | **0.2360** | **0.2017** | **2** | **3.282** |
| **16** | **D041** | **28** | **28** | **0.3402** | **0.1937** | **0.1921** | **2** | **5.719** |
| **17** | **D048** | **37** | **35** | **0.3021** | **0.2029** | **0.1834** | **1** | **0.657** |
| **18** | **D060** | **29** | **29** | **0.2880** | **0.1753** | **0.2015** | **2** | **6.719** |
| **19** | **D065** | **26** | **24** | **0.3513** | **0.2038** | **0.2339** | **2** | **3.719** |
| **20** | **D067** | **27** | **25** | **0.3366** | **0.1949** | **0.2356** | **2** | **4.719** |
| **21** | **D069** | **21** | **19** | **0.3107** | **0.2523** | **0.2115** | **2** | **1.282** |
| **22** | **D073** | **40** | **40** | **0.2673** | **0.2133** | **0.2124** | **1** | **3.655** |
| **23** | **D076** | **43** | **41** | **0.3422** | **0.2468** | **0.2458** | **1** | **6.655** |
| **24** | **D078** | **25** | **23** | **0.3449** | **0.1941** | **0.2926** | **2** | **2.720** |
| **25** | **D080** | **35** | **33** | **0.2600** | **0.2043** | **0.2018** | **1** | **1.348** |
| **26** | **D081** | **33** | **31** | **0.2635** | **0.1818** | **0.2134** | **1** | **3.345** |
| **27** | **D082** | **45** | **43** | **0.3009** | **0.2267** | **0.2215** | **1** | **8.655** |
| **28** | **D083** | **21** | **21** | **0.3239** | **0.2119** | **0.2222** | **2** | **1.281** |
| **29** | **D084** | **39** | **37** | **0.2604** | **0.2321** | **0.2188** | **1** | **2.655** |
| **30** | **D088** | **36** | **35** | **0.3054** | **0.2150** | **0.2211** | **1** | **0.348** |
| **31** | **D089** | **19** | **19** | **0.2718** | **0.2045** | **0.2451** | **2** | **3.281** |
| **32** | **D092** | **19** | **17** | **0.2384** | **0.2011** | **0.1890** | **2** | **3.282** |
| **33** | **D093** | **20** | **20** | **0.3131** | **0.2016** | **0.2382** | **2** | **2.282** |
| **34** | **D098** | **42** | **40** | **0.2939** | **0.2042** | **0.1996** | **1** | **5.655** |
| **35** | **D099** | **41** | **40** | **0.3049** | **0.2302** | **0.2124** | **1** | **4.655** |
| **36** | **D106** | **34** | **34** | **0.3093** | **0.1671** | **0.1910** | **1** | **2.345** |
| **37** | **D108** | **19** | **18** | **0.3070** | **0.2124** | **0.2127** | **2** | **3.282** |
| **38** | **D110** | **36** | **35** | **0.2805** | **0.1852** | **0.2423** | **1** | **0.345** |
| **39** | **D113** | **27** | **26** | **0.2951** | **0.1963** | **0.2210** | **2** | **4.719** |
| **40** | **D116** | **21** | **20** | **0.3185** | **0.2612** | **0.2640** | **2** | **1.281** |
| **41** | **D119** | **31** | **30** | **0.3051** | **0.1813** | **0.1781** | **1** | **5.345** |
| **42** | **D120** | **32** | **30** | **0.3060** | **0.1878** | **0.1942** | **1** | **4.345** |
| **43** | **D121** | **19** | **18** | **0.2845** | **0.2324** | **0.2132** | **2** | **3.281** |
| **44** | **D124** | **18** | **16** | **0.3201** | **0.2227** | **0.2160** | **2** | **6.281** |
| **45** | **D125** | **22** | **20** | **0.3401** | **0.2084** | **0.2329** | **2** | **0.282** |
| **46** | **D129** | **32** | **32** | **0.2996** | **0.2095** | **0.2089** | **1** | **4.345** |
| **47** | **D131** | **31** | **30** | **0.3001** | **0.1807** | **0.2320** | **1** | **5.345** |
| **48** | **D132** | **40** | **40** | **0.2879** | **0.1966** | **0.2122** | **1** | **3.655** |
| **49** | **D133** | **18** | **16** | **0.3425** | **0.1935** | **0.2060** | **2** | **4.281** |
| **50** | **D135** | **23** | **22** | **0.3077** | **0.1792** | **0.2329** | **2** | **0.720** |
| **51** | **D136** | **25** | **24** | **0.3488** | **0.2241** | **0.2396** | **2** | **2.719** |
| **52** | **D143** | **28** | **26** | **0.3292** | **0.1891** | **0.2195** | **2** | **5.719** |
| **53** | **D144** | **29** | **27** | **0.2939** | **0.1992** | **0.1874** | **2** | **6.719** |
| **54** | **D145** | **38** | **37** | **0.2845** | **0.2136** | **0.2113** | **1** | **1.656** |
| **55** | **D146** | **34** | **32** | **0.3084** | **0.1882** | **0.2299** | **1** | **2.345** |
| **56** | **D147** | **18** | **16** | **0.3054** | **0.2420** | **0.2458** | **2** | **4.281** |
| **57** | **D148** | **21** | **19** | **0.2450** | **0.2130** | **0.2387** | **2** | **1.282** |
| **58** | **D153** | **23** | **22** | **0.2448** | **0.2296** | **0.2241** | **2** | **0.722** |
| **59** | **D155** | **34** | **33** | **0.2870** | **0.2024** | **0.2009** | **1** | **2.346** |
| **60** | **D162** | **20** | **19** | **0.3460** | **0.2091** | **0.2524** | **2** | **2.281** |
| **61** | **D166** | **31** | **30** | **0.2736** | **0.1770** | **0.1992** | **1** | **5.345** |

RTEMP, RMFG and LOCG represent significant clusters in MDD differed from HCs (showed in supplementary figure 2).

**Table S3 Abnormal FA clusters in EO and LO subgroups of MDD**

|  | **Anatomical Region** | **Nearest GM** |  | **Z** | **Voxel size** | **x** | **y** | **z** |
| --- | --- | --- | --- | --- | --- | --- | --- | --- |
| ***EO*** | |  |  |  |  |  |  |  |
|  | ***MDD<HCs*** |  |  |  |  |  |  |  |
|  | **Temporal Lobe** |  |  |  |  |  |  |  |
| **b** | **Inferior longitudinal fasciculus**  **Inferior fronto-occipital fasciculus** | **Parahippocampal Gyrus, BA36**  **Superior Temporal Gyrus, BA21** | **L** | **2.97** | **853** | **-42**  **-51** | **-31**  **-26** | **-10**  **-3** |
|  | ***MDD>HCs*** |  |  |  |  |  |  |  |
|  | **Midbrain** |  |  |  |  |  |  |  |
| **a** | **Corticospinal/ corticopontine tract** | **Substantia Nigra** | **R** | **3.14** | **618** | **11** | **-22** | **-16** |
|  | **Occipital Lobe** |  |  |  |  |  |  |  |
| **c** | **Forceps major**  **Optical Radiation** | **Cuneus, BA17**  **Middle Occipital Gyrus, BA18** | **R** | **2.85** | **2136** | **20**  **13** | **-81**  **-89** | **8**  **17** |
| **d** | **Inferior longitudinal fasciculus**  **Inferior fronto-occipital fasciculus** | **Superior Occipital Gyrus, BA19** | **L** | **3.56** | **2367** | **-30** | **-78** | **26** |
|  | **Cingulate** |  |  |  |  |  |  |  |
| **e** | **Corpus callosum** | **Posterior Cingulate Gyrus, BA31**  **Cuneus, BA7** | **R** | **3.37** | **2128** | **20**  **17** | **-62**  **-71** | **17**  **31** |
| **f** | **Corpus callosum** | **Cingulate Gyrus, BA31** | **L** | **2.82** | **991** | **-14** | **-41** | **37** |
| ***LO*** | |  |  |  |  |  |  |  |
|  | ***MDD<HCs*** |  |  |  |  |  |  |  |
|  | **Frontal Lobe** |  |  |  |  |  |  |  |
| **A** | **Inferior fronto-occipital fasciculus**  **Anterior corona radiata** | **Orbitales Gyrus**  **Inferior Frontal Gyrus, BA11** | **L** | **4.05** | **1612** | **-18** | **25** | **-19** |
| **B** | **Inferior fronto-occipital fasciculus**  **Anterior thalamic radiation** | **Orbitales Gyrus**  **Inferior Frontal Gyrus, BA11** | **R** | **3.05** | **1378** | **20**  **23** | **32**  **39** | **-19**  **-14** |
|  | **Temporal Lobe** |  |  |  |  |  |  |  |
| **C** | **Inferior longitudinal fasciculus** | **Inferior Temporal Gyrus, BA37** | **R** | **3.57** | **2577** | **44** | **-47** | **-8** |
|  | **Occipital Lobe** |  |  |  |  |  |  |  |
| **D** | **Inferior fronto-occipital fasciculus**  **Inferior longitudinal fasciculus** | **Middle Occipital Gyrus, BA19** | **R** | **3.03** | **723** | **35** | **-77** | **5** |
|  | **Thalamus** |  |  |  |  |  |  |  |
| **E** | **Posterior limb of internal capsule**  **Superior thalamic radiation** | **Nucleus caudate**  **Cingulate Gyrus, BA24** | **L** | **3.36** | **3803** | **-15**  **-5** | **0**  **-13** | **23**  **16** |
|  | **Parietal Lobe** |  |  |  |  |  |  |  |
| **F** | **Superior thalamic radiation** | **Precuneus, BA5**  **Cingulate Gyrus, BA31** | **R** | **2.91** | **707** | **13**  **21** | **-41**  **-34** | **53**  **40** |
| **G** | **Posterior corona radiata** | **Precuneus/Superior Parietal Gyrus, BA7** | **R** | **3.57** | **1080** | **15** | **-59** | **54** |

Small letters a to f, clusters with FA differences between EO subgroup of MDD and HCs. Capital letters A to G, clusters with FA difference between LO subgroup of MDD and HCs. L, left hemisphere; R, right hemisphere. GM, grey matter. BA, Brodmann area.

**Table S4 Abnormal FA clusters in MDD patients at the age of 18-25 years old compared with paired HCs**

|  | | **Anatomical Region** | **Nearest GM** |  | **Z** | **Voxel**  **size** | **x** | **y** | **z** |
| --- | --- | --- | --- | --- | --- | --- | --- | --- | --- |
| ***MDD>HCs*** | | |  |  |  |  |  |  |  |
|  | **Midbrain** | |  |  |  |  |  |  |  |
| **1, b** | **Corticospinal/ corticopontine tract** | | **Substantia Nigra** | **R** | **3.14** | **970** | **6** | **-20** | **-15** |
|  | **Frontal lobe** | |  |  |  |  |  |  |  |
| **2** | **Inferior fronto-occipital fasciculus**  **Anterior thalamic radiation** | | **Orbitales Gyrus**  **Inferior/Middle Frontal Gyrus, BA10** | **R** | **2.87** | **913** | **14** | **50** | **-5** |
| **3** | **Inferior fronto-occipital fasciculus**  **Anterior thalamic radiation** | | **Orbitales Gyrus**  **Inferior/Middle Frontal Gyrus, BA10** | **L** | **3.55** | **1810** | **-35** | **47** | **-2** |
| **6** | **Inferior fronto-occipital fasciculus** | | **Insula, BA13** | **L** | **3.05** | **817** | **-39** | **12** | **22** |
|  | **Occipital Lobe** | |  |  |  |  |  |  |  |
| **4,c** | **Forceps major**  **Optical Radiation** | | **Cuneus, BA17**  **Middle Occipital Gyrus, BA18** | **R** | **3.57** | **1968** | **21** | **-82** | **7** |
| **5,e** | **Inferior longitudinal fasciculus**  **Inferior fronto-occipital fasciculus** | | **Superior Occipital Gyrus, BA19** | **L** | **3.87** | **2890** | **-30** | **-80** | **27** |
|  | **Parietal lobe** | |  |  |  |  |  |  |  |
| **7** | **Posterior corona radiata**  **Superior longitudinal fasciculus** | | **Precuneus, BA7**  **Inferior Parietal Lobule, BA40** | **R** | **2.93** | **693** | **37** | **-50** | **33** |

**Table S5 FA Clusters correlated with HDRS scores in EO and LO subgroups of MDD**

|  | **Anatomical Region** | **Nearest GM** |  | **Z** | **Voxel size** | **x** | **y** | **z** |
| --- | --- | --- | --- | --- | --- | --- | --- | --- |
| ***EO*** |  |  |  |  |  |  |  |  |
| ***Positive correlation*** | |  |  |  |  |  |  |  |
|  | **Midbrain** |  |  |  |  |  |  |  |
| **g** | **Corticospinal/ corticopontine tract** | **Substantia Nigra** | **L** | **3.50** | **1017** | **-10** | **-18** | **-10** |
|  | **Temporal Lobe** |  |  |  |  |  |  |  |
| **h** | **Inferior longitudinal faciculus** | **Inferior Temporal Gyrus, BA20**  **Fusiform Gyrus, BA20**  **Parahippocampal Gyrus, BA36** | **L** | **2.99** | **677** | **-43**  **-43**  **-42** | **-18**  **-24**  **-36** | **-25**  **-15**  **-7** |
| ***Negative correlation*** | |  |  |  |  |  |  |  |
|  | **Temporal Lobe** |  |  |  |  |  |  |  |
| **i** | **Inferior longitudinal/ Fronto-occipital faciculus** | **Middle Temporal Gyrus, BA39** | **L** | **4.00** | **2757** | **-39** | **-56** | **6** |
|  | **Parietal Lobe** |  |  |  |  |  |  |  |
| **j** | **Superior longitudinal fasciculus** | **Inferior Parietal Lobule, BA40** | **L** | **3.75** | **1867** | **-40** | **-42** | **39** |
|  | **Frontal lobe** |  |  |  |  |  |  |  |
| **k** | **Superior longitudinal fasciculus** | **Middle Frontal Gyrus, BA6** | **R** | **3.71** | **608** | **30** | **9** | **40** |
| ***LO*** |  |  |  |  |  |  |  |  |
| ***Negative correlation*** | |  |  |  |  |  |  |  |
|  | **Limbic Lobe** |  |  |  |  |  |  |  |
| **H** | **Cingulum** | **Uncus, BA20**  **Parahippocampal Gyrus, BA36** | **R** | **3.35** | **756** | **35**  **27** | **-16**  **-19** | **-28**  **-28** |
|  | **Frontal lobe** |  |  |  |  |  |  |  |
| **I** | **Inferior fronto-occipital fasciculus** | **Orbitales Gyrus /Inferior Frontal Gyrus, BA47** | **L** | **3.20** | **1172** | **-22** | **11** | **-14** |
| **J** | **Uncinate fasciculus**  **Inferior fronto-occipital fasciculus** | **Orbitales Gyrus /Middle Frontal Gyrus, BA11** | **R** | **4.08** | **2404** | **27**  **40** | **40**  **35** | **-15**  **-8** |
| **K** | **Anterior corona radiata**  **Superior fronto-occipital fasciculus** | **Inferior Frontal Gyrus, BA46**  **Medial Frontal Gyrus, BA9** | **R** | **3.55** | **3891** | **36**  **19** | **30**  **29** | **11**  **24** |
| **L** | **External capsule**  **Inferior fronto-occipital fasciculus** | **Insula**  **Middle Frontal Gyrus, BA9** | **L** | **3.07** | **1106** | **-34**  **-28** | **20**  **20** | **16**  **26** |
| **M** | **Superior longitudinal fasciculus** | **Precentral Gyrus, BA6** | **R** | **3.32** | **1196** | **38** | **-9** | **34** |
|  | **Thalamus** |  |  |  |  |  |  |  |
| **N** | **Anterior limb of internal capsule** | **Caudate** | **R** | **3.58** | **1234** | **10** | **13** | **18** |
| **O** | **Fornix** | **Thalamus** | **L** | **3.73** | **1478** | **0** | **-18** | **18** |
|  | **Parietal Lobe** |  |  |  |  |  |  |  |
| **P** | **Posterior corona radiata**  **Superior longitudinal fasciculus** | **Precuneus, BA7**  **Inferior Parietal Lobule, BA40** | **R** | **3.06** | **1416** | **28**  **31** | **-48**  **-44** | **45**  **33** |

Small letters from g to k, FA clusters correlated with HDRS scores in EO subgroup of MDD. Capital letters from H to P, clusters which the FA value correlated with HDRS scores in LO subgroup of MDD.
