# Supplementary material for: Delineation of Early and Later Adult Onset Depression by Diffusion Tensor Imaging
Source: PLoS One. 2014 Nov 13;9(11):e112307. doi: 10.1371/journal.pone.0112307 (PMC4231105; doi:10.1371/journal.pone.0112307)
Supplement: Protocol S1 — Trial protocol. (DOCX) [file pone.0112307.s003.docx]

Protocol S1

A Neuroimaging Investigation of Antidepressants in Depression

**1.Backgroud**

**2.Review of previous studies**

**3. Main object**

a. to find out the structural or functional effects of selective serotonin reuptake inhibitors (SSRI) in major depressive disorder (MDD);

b. to find special abnormalities in depression secondary to other disease, e.g., autoimmune disease like systemic lupus erythematosus (SLE).

c. to find the relationship between the efficacy of antidepressant and the change of neuroimaging in MDD

d. to find possible predispose to MDD

e. to explore the DNA methylation status in depression;

**4.Methods**

(1)procedure：

MDD: inform consent evaluate blood sample MRI scan antidepressant treatment MRI rescan

control: inform consent evaluate blood sample MRI scan

(2) Criteria：

Inclusion Criteria:

DSM-IV， Major Depressive Disorder，SCID

Age 18-65

Physically healthy

Drug-free

Exclusion Criteria:

Body metal (e.g., wire stitches, screws in bones, stainless steel hips)

History of Psychosis or Epilepsy

Current (past six months) Substance Use Disorder (illicit drugs and/or alcohol)

Bipolar I

Need for wash-out from effective treatment in order to participate

Pregnant

High suicide risk

Currently taking (within 2 weeks; 4 weeks for Fluoxetine) antidepressants

Bad compliance

Sample size: 50 MDD

(3)Treatment: Escitaropram 10mg/day, 8 weeks

(4) Scales: HAMD（MADRS）,HAMA,STAI,CGI,TESS,LES

time: baseline,1 week, 2 week, 4week, 8week

(5)Blood sample: 3ml, －70℃

time: baseline, 8week

(6)MRI: 3D,fMRI,DTI

time: baseline, 8week

Control group: matched with age, sex, education. without physical problem and psychiatric disorder.

**5.statistics：**

correlation, factor analyze, ANOVA

**6. Novelty**

a.to compare the acute and chronic effect of antidepressant on brain

b.to compare the difference of brain structure between responder and non-responder

**7. Anticipated progress**

2008/08-2011/07：recruiting

2011/8-2012/08：analysis, statistics

2012/8-2013/08：compose articles
